# Effects of different food ingredients on the color and absorption spectrum of carminic acid and carminic aluminum lake

**DOI:** 10.1002/fsn3.1628

**Published:** 2020-12-03

**Authors:** Qian Liu, Zhiyong He, Maomao Zeng, Fang Qin, Zhaojun Wang, Guoping Liu, Jie Chen

**Affiliations:** ^1^ State Key Laboratory of Food Science and Technology Jiangnan University Wuxi China; ^2^ International Joint Laboratory on Food Safety Jiangnan University Wuxi China; ^3^ Department of Endocrinology Wuxi People's Hospital Wuxi China

**Keywords:** carminic acid, carminic aluminum lake, color, food additives, metal ions, protein

## Abstract

In this study, three foodstuffs (surimi, minced meat, and milk) were dyed with carminic acid and carminic aluminum lake. The effects of protein, metal ions, and food additives on the color of carminic acid and carminic aluminum lake were investigated. After being dyed by carminic acid, the colors of surimi, minced meat, and milk were light purple, red, and gray‐green, respectively. When using carminic aluminum lake, surimi and milk were magenta, and minced meat was red. Regarding the carminic acid solution, the presence of myofibrillar protein (MFP), whey protein isolate (WPI), and soy protein isolate (SPI) turned it red by changing the pH, while the presence of casein made it orange. The carminic aluminum lake solution turned magenta in all four cases, which were not affected by protein. The color of carminic acid and carminic aluminum lake was significantly affected by 0.001–0.1 mol/L Fe^3+^, 0.001–0.1 mol/L Fe^2+^, 0.001–0.1 mol/L Cu^2+^, and 0.1 mol/L Ca^2+^, limiting their application in iron‐, copper‐, and high‐calcium foods. The color of carminic acid was changed to yellow by 0.01%‐1% sodium nitrite, but 0.01%–1% ascorbic acid and 0.01%–0.1% monascus color did not significantly affect the color of either carminic acid or carminic aluminum lake. This paper provides a reference for the application of carminic acid and carminic aluminum lake in food science.

## Graphical abstract

Three foodstuffs (surimi, minced meat and milk) were dyed by carminic acid and carminic aluminum lake. The effects of protein, metal ions and food additives on the color of carminic acid and carminic aluminum lake were investigated.This paper provides a reference for the application of carminic acid and carminic aluminum lake in food industry.

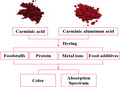

## 1 INTRODUCTION

Color is an important indicator for accurately evaluating food quality in nutrition science. The color tests have a great effect on the degree of acceptance of foods by consumers. Nowadays, to color food in a hue that is easily accepted by consumers, a colorant is usually used, which may be natural or artificial. Natural colorants are extracted from animals, plants, and minerals by solvent extraction, microwave, ultrasonic, supercritical extraction, etc (Borges, Tejera, Díaz, Esparza, & Ibáñez, 2012; González, Méndez, Carnero, Lobo, & Afonso, 2002; Zia et al., 2019; Zuber et al., 2019). As compared to artificial colorants, natural ones are safer and have documented nutritional and pharmacological effects (Matsui et al., 2002; Nistor et al., 1990). Hence, researchers are more interested in the use of natural colorants.

Carmine is a natural anthraquinone colorant that is extracted from Cochineal (Dactylopius coccus L. Costa) (Stathopoulou, Valianou, Skaltsounis, Karapanagiotis, & Magiatis, 2013) and has good thermal and light stability. It has been used for dyeing cosmetics and textiles (Adeel et al., 2019; Amin et al., 2020; Mendez‐Gallegos, Panzavolta, & Tiberi, 2003) and has received allowance to be used as a food colorant by the Food and Agriculture Organization of the United Nations (FAO), the World Health Organization, and the US Food and Drug Administration (Borges et al., 2012). Carmine can be divided into carminic acid and carminic aluminum lake. Carminic acid is a β‐C‐glycopyranosyl derivative of anthraquinone. Its chromophore existed in the anthracycline and its chromogenic properties depended on the number and position of the hydroxy group (Miliani, Romani, & Favaro, 2000). Carminic acid has very interesting photoactivity, and it shows different colors in different environments, affecting its application in different food systems. For example, its color can change from orange to purple with its pH increasing from 2 to 12 (Favaro, Miliani, Romani, & Vagnini 2002); it can turn taupe when it is exposed to Fe^3+^ or Ca^2+^ and can turn purple when it is exposed to protein (Wang, Yu, & Wang, 2007). At present, the colorant that is commonly used to dye food is carminic aluminum lake, a bi‐coordinated complex with a six‐membered chelate ring structure that is formed by the aluminum atom chelated with two molecules of carmine acid via the 5‐hydroxyl group and the ortho‐carbonyl oxygen (Favaro et al. 2002). Compared with carminic acid, the color of carminic aluminum lake is more stable under complex conditions (Mendez‐Gallegos et al., 2003; Pozzi, van den Berg, Fiedler, & Casadio, 2014). However, due to the presence of aluminum, the safety of its application in food is still debated (Ding et al., 2019; Yokel, 2006; Zhu et al., 2011).

The system of food is very complicated, and the color of the colorant depends on the existence of the conjugated unsaturated system in the dye molecule, so any substance in the food sample that can change the system may affect its color (Scotter & Castle, 2004).

For the purpose of knowing more clearly about the color of carminic acid and carminic aluminum lake in different food systems and the effects of different components in the food on its color, we selected surimi, minced meat, and milk to be dyed and discussed the effects of protein, metal ions, and other food additives in the system on the color of carminic acid and carminic aluminum lake in this paper, to provide a reference for the further application of carminic acid and carminic aluminum lake in food.

## 2 MATERIALS AND METHODS

### 2.1 Materials

Sea bass, pork tenderloin, and soybean (Taiwan 292) were purchased from a local supermarket. Milk was purchased from Mengniu Dairy Co., Ltd. Carminic acid (~85%) and carminic aluminum lake were obtained from Tongyi Biological Technology Co., Ltd. Whey protein isolate (WPI) (~92%) was purchased from Davisco Foods International Inc. Casein was purchased from Fonterra Co‐operative Group. Monascus color was obtained from Yinuo Biotechnology Co., Ltd. Disodium phosphate dodecahydrate, citric acid, iron (III) chloride hexahydrate, ferrous chloride tetrahydrate, anhydrous calcium chloride, copper (II) sulfate pentahydrate, sodium chloride, potassium chloride, sodium nitrite, and ascorbic acid were purchased from Sinopharm Chemical Reagent Co., Ltd.

### 2.2 Dyeing of different foodstuffs

After removing the fish bones, the fish and pork tenderloin were ground into surimi and minced meat using a tissue masher. Carminic acid was added to surimi, minced meat, and milk at a mass ratio of 1:4,500, 1:4,500, and 1:2,000, respectively. These samples were stirred evenly, and carminic aluminum lake was used in the same proportion to dye surimi, minced meat, and milk. The color and pH of surimi, minced meat, and milk before and after adding carminic acid and carminic aluminum lake were recorded. According to GB/T 9695.5–2008, when the pH of surimi and minced meat was measured, ten times the sample volume of 0.1 mol/L KCl was added to the sample and dispersed with a disperser homogenizer (T 18 basic ULTRA‐TURRAX®, IKA Corp).

### 2.3 Effects of myofibrillar protein (MFP), whey protein isolate (WPI), soy protein isolate (SPI), and casein on the colorants

Myofibrillar protein was extracted from pork tenderloin according to the method described by Yu et al. (2016), and the protein content was ~ 80.65 mg/ml determined by Biuret method. SPI was prepared from soybean according to the method described by Guo et al. (2015), and the protein content was ~87%, as determined by Kjeldahl's method.

A quantity of 0.2 mol/L disodium hydrogen phosphate and 0.1 mol/L citric acid were mixed to prepare buffer solutions with a pH of 6 and 8, respectively. Carminic acid and water were mixed at a mass ratio of 1:5,000 to prepare the colorant solution. WPI, SPI, and casein were added to the colorant solution at 1% (w/w) protein content, respectively, and they were stirred evenly. MFP was added to the colorant solution to make the protein content reach 5.38 mg/mL and stirred evenly. According to the above steps, the above proteins were added to the colorant solution at a pH of 6 and 8, respectively. The color, pH, and absorption spectrum of carminic acid before and after adding protein were recorded.

The carminic acid was replaced by carminic aluminum lake, and the above steps were repeated.

### 2.4 Effects of metal ions on the colorants

Carminic acid and water were mixed at a mass ratio of 1:5,000 to prepare the colorant solution. FeCl_3_, FeCl_2_, CaCl_2_, CuSO_4_ were added to colorant solution at concentrations of 0.001 mol/L and 0.1 mol/L, respectively, and they were stirred evenly. The color, pH, and absorption spectrum of carminic acid before and after adding metal ions were recorded.

The carminic acid was replaced by carminic aluminum lake, and the above steps were repeated.

### 2.5 Effects of food additives on the colorants

Carminic acid and water were mixed at a mass ratio of 1:5,000 to prepare colorant solution. Sodium nitrite and ascorbic acid were added to colorant solution at a mass concentration of 0.01% and 1%, respectively, and they were stirred evenly. Monascus color was added to colorant solution at a mass concentration of 0.01% and 0.1%, respectively, and stirred evenly. The color, pH, and absorption spectrum of carminic acid before and after adding metal ions were recorded.

The carminic acid was replaced by carminic aluminum lake, and the above steps were repeated.

### 2.6 Sample measurement and statistical analysis

The color was recorded by camera. The pH was measured by pH meter (Seven Easy, Mettler Toledo). The absorption spectrum was measured by enzyme‐labeled instrument (Spectramax 190, Molecular Devices) between 190 nm and 750 nm.

Each measurement was performed at least three times, and the results are expressed as means ± standard deviation. The data were mapped with OriginPro 2017. Statistics 9.0 was used to analyze an analysis of variance (ANOVA).

## 3 RESULTS AND DISCUSSION

### 3.1 Dyeing of different foodstuffs

**Figure 1:**
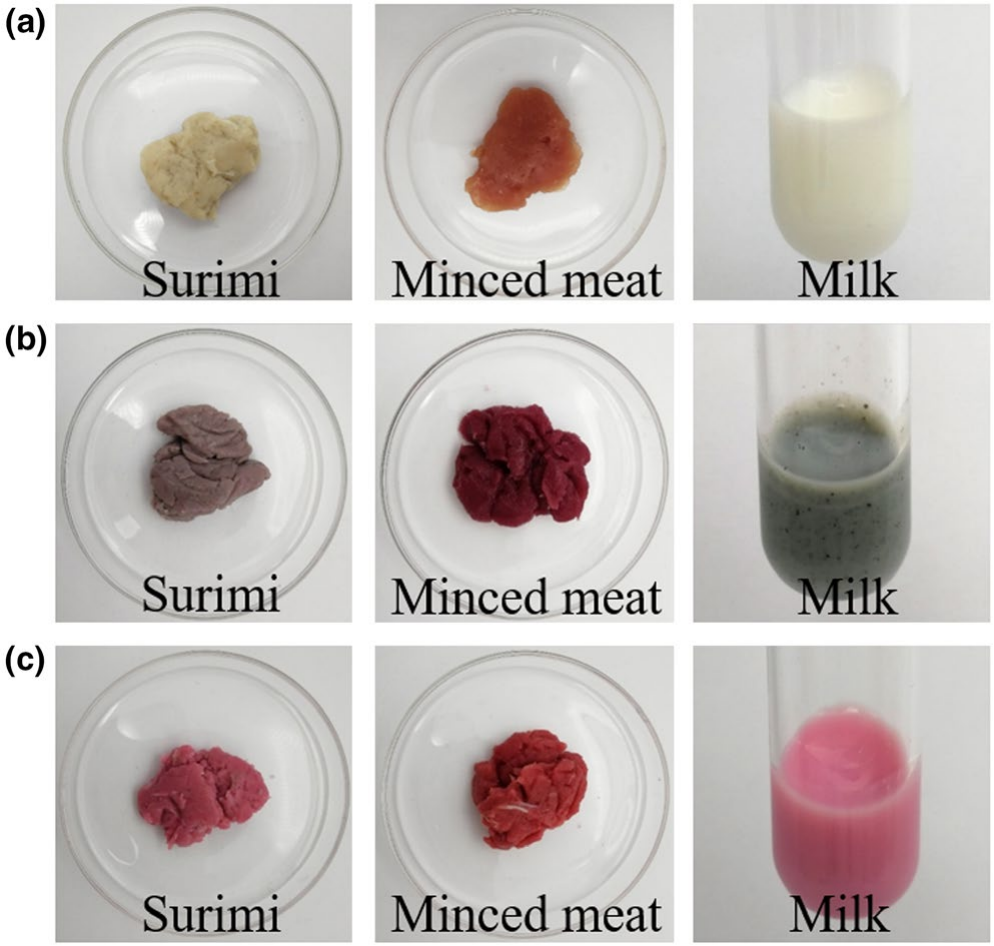
Color of surimi, minced meat, and milk before and after being dyed by carminic acid and carminic aluminum lake. (a) Blank, (b) carminic acid, (c) carminic aluminum lake

**Figure 2:**
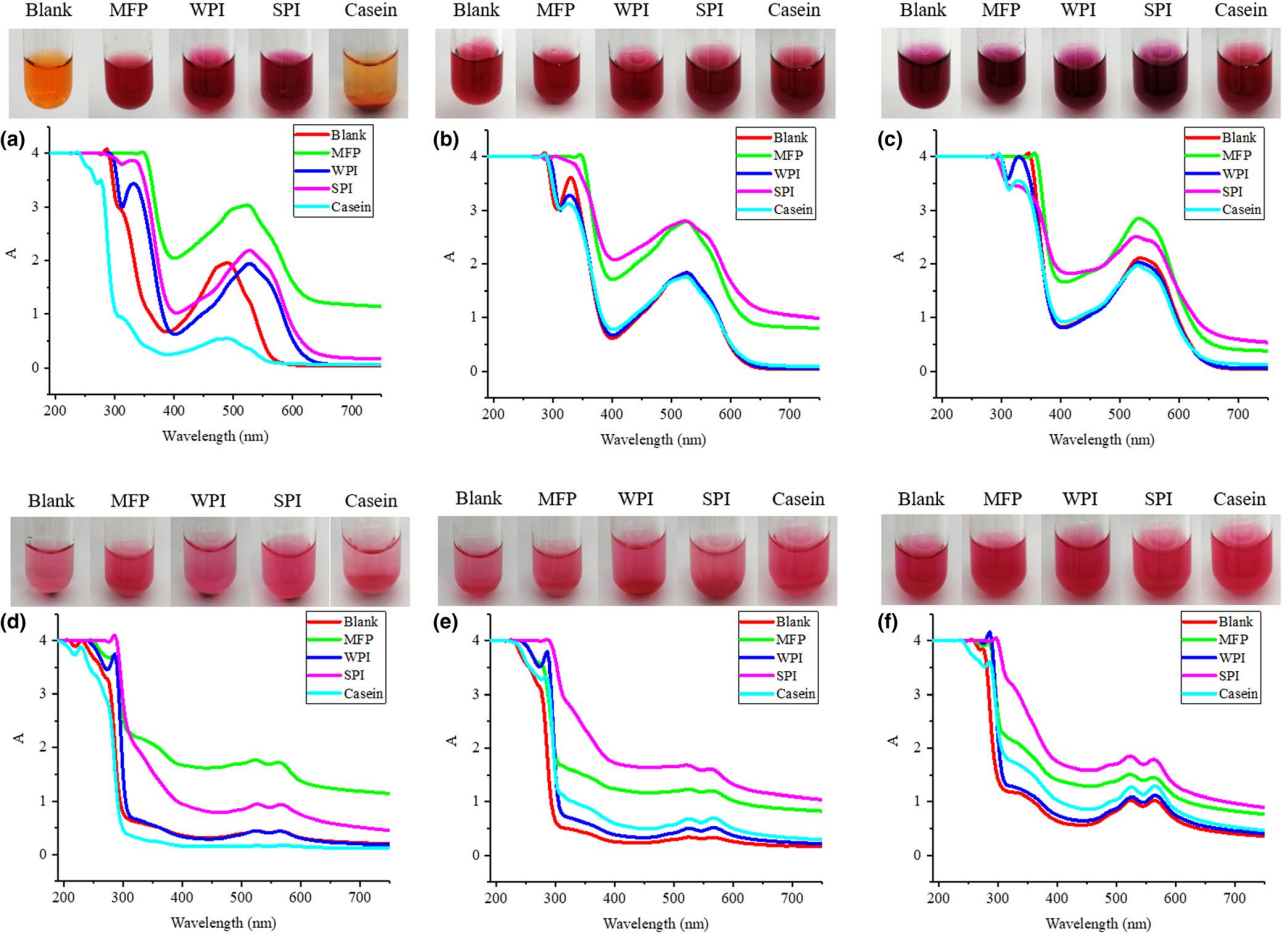
Effects of MFP, WPI, SPI, and casein on the color and absorption spectrum of carminic acid and carminic aluminum lake at different pH. (a) Water, carminic acid (b) pH 6, carminic acid (c) pH 8, carminic acid (d) Water, carminic aluminum lake (e) pH 6, carminic aluminum lake (f) pH 8, carminic aluminum lake

Surimi, minced meat, and milk are typical food systems that need to be dyed by colorants. Figure 1 shows the color of surimi, minced meat, and milk before and after being dyed by carminic acid and carminic aluminum lake. According to Figure 1 and Table 1, the surimi turned purple after being dyed by carminic acid, which may be caused by its pH of 6.61 and the presence of iron element. The minced meat was dark red after being dyed with carminic acid, possibly due to the presence of MFP in the minced meat, which was confirmed by the color of carminic acid mixed with MFP as shown in Figure 2(a). When carminic acid was added to milk, it turned taupe, which may be caused by the presence of calcium ions in milk. In contrast, the color of carminic aluminum lake was more stable. After being dyed by carminic aluminum lake, surimi and milk were magenta and minced meat was red. This may be due to the chelation of Al^3+^ with the hydroxyl group at the 5‐position and the ortho‐carbonyl oxygen in the carminic aluminum lake (Harris, Stein, Tyman, & Williams, 2009), which makes intramolecular protons less likely to migrate and allows the color of carminic aluminum lake to be less affected by the surrounding environment.

**Table 1:** The pH of surimi, minced meat, and milk before and after being dyed by carminic acid and carminic aluminum lake

Conditions	Surimi	Minced meat	Milk
Blank	6.61 ± 0.01a	6.63 ± 0.02a	6.73 ± 0.02a
Carminic acid	6.54 ± 0.04b	6.55 ± 0.01b	6.52 ± 0.03c
Carminic aluminum lake	6.56 ± 0.02ab	6.63 ± 0.03a	6.65 ± 0.01b

Note

Data are expressed as the mean ± *SD* (*n* = 3). The different letters (a–b) in the same column indicate significant difference among the values at the *p* < .05.

### 3.2 Effects of MFP, WPI, SPI, and casein on the colorants

There is a high content of MFP, WPI, and casein in meat and dairy products, respectively. SPI can be used as a food additive in meat products. The presence of MFP, WPI, SPI, and casein may affect the application of carminic acid and carminic aluminum lake in meat and dairy products.

Figure 2 shows the effects of MFP, WPI, SPI, and casein on the color and absorption spectrum of carminic acid and carminic aluminum lake. According to Figure 2 and Table 2, after added with MFP, WPI, and SPI, the carminic acid aqueous solutions were all red and their pH increased to 6.09, 6.77, and 7.04, respectively. Their maximum absorption wavelength was all 530 nm, which was consistent with the maximum absorption wavelength of carminic acid solution at a pH of 6. This shows that the change in the color of the solution may be caused by the presence of amino groups in the protein, which changed the pH of the solution. In this environment, protonated amino groups in the protein and negatively charged carminic acid may form a complex through electrostatic interactions (Sun, Han, & Jiao, 2006). While controlling the pH, the color of the carminic acid solution and the absorption spectrum were not significantly changed after adding the protein, which further confirms this idea. Due to the low solubility of casein in the aqueous solution, the pH of the carminic acid aqueous solution was 3.92 and the color was orange after the casein was added. In contrast, the color of carminic aluminum lake after adding protein was all magenta and has not been changed. This shows that the effect of electrostatic interaction on the carminic aluminum lake is weaker than the chelation of Al^3+^, which is not enough to affect its color.

**Table 2:** Effects of MFP, WPI, SPI, and casein on the pH of carminic acid and carminic aluminum lake

Conditions		Blank	MFP	WPI	SPI	Casein
Carminic acid	Water	3.40 ± 0.02c	6.09 ± 0.02b	6.77 ± 0.01a	7.04 ± 0.01a	3.92 ± 0.02c
	pH6	6.08 ± 0.02a	6.07 ± 0.00a	6.06 ± 0.00a	6.07 ± 0.00a	5.97 ± 0.02b
	pH8	8.03 ± 0.01a	7.98 ± 0.01b	7.95 ± 0.00c	7.96 ± 0.00c	7.73 ± 0.00d
Carminic aluminum lake	Water	5.50 ± 0.07d	6.62 ± 0.02c	7.21 ± 0.04b	7.45 ± 0.01a	3.99 ± 0.04e
	pH6	6.07 ± 0.01ab	6.10 ± 0.01a	6.06 ± 0.00b	6.10 ± 0.00a	5.98 ± 0.02c
	pH8	8.03 ± 0.00a	8.01 ± 0.00b	7.97 ± 0.00d	8.00 ± 0.00c	7.76 ± 0.01e

Note

Data are expressed as the mean ± *SD* (*n* = 3). The different letters (a–e) in the same row indicate significant difference among the values at the *p* < .05.

### 3.3 Effects of metal ions on the colorants

**Figure 3:**
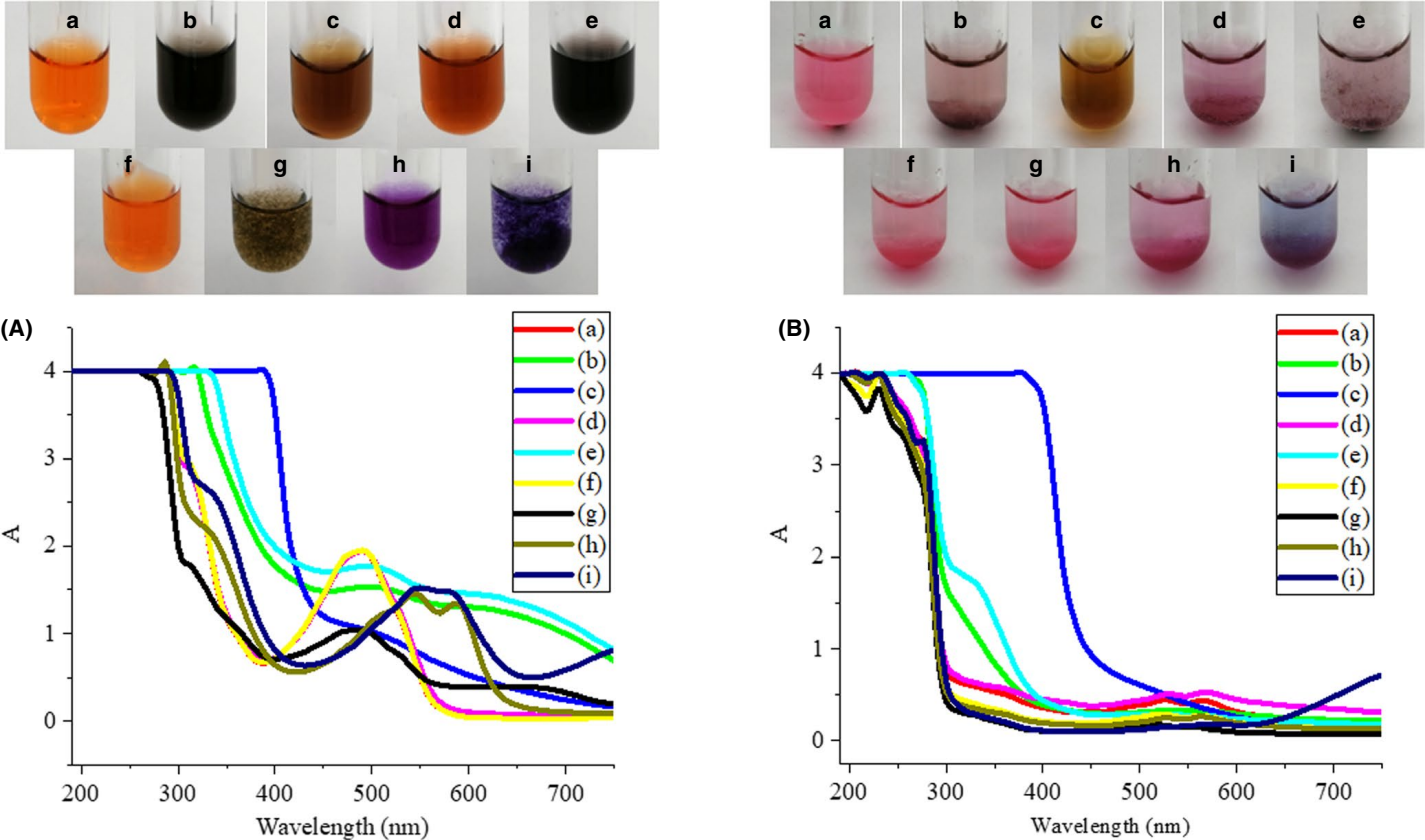
Effects of FeCl_3_, FeCl_2_, CaCl_2_, and CuSO_4_ at different concentrations on the color and absorption spectrum of carminic acid (A) and carminic aluminum lake (B). (a) Blank (b) 0.001 mol/L FeCl_3_ (c) 0.1 mol/L FeCl_3_ (d) 0.001 mol/L FeCl_2_ (e) 0.1 mol/L FeCl_2_ (f) 0.001 mol/L CaCl_2_ (g) 0.1 mol/L CaCl_2_ (h) 0.001 mol/L CuSO_4_ (i) 0.1 mol/L CuSO_4_

Meat and dairy products often contain some metal ions such as Fe^3+^, Fe^2+^, Ca^2+^, Cu^2+^. Previous studies have shown that metal ions such as iron and copper can be chelated with carminic acid to form metal complexes (Atabey, Sari, & Al‐Obaidi, 2012; Tütem, Apak, & Zgen, 1996), which can affect the color of carminic acid and limit the use of it. The color of carminic acid is closely related to its absorption spectrum. Figure 3 shows the effects of FeCl_3_, FeCl_2_, CaCl_2_, and CuSO_4_ at different concentrations on the color and absorption spectrum of carminic acid and carminic aluminum lake.

It can be seen from Figure 3(a) that when the concentration of Fe^3+^ was 0.001 mol/L, the carminic acid solution was black, the carminic aluminum lake solution was light purple, and they both had weak absorption peaks at 530 nm. When the concentration of Fe^3+^ increased to 0.1 mol/L, the carminic acid solution was brown, the carminic aluminum lake solution was brown‐yellow, and there were no obvious absorption peaks in the visible light region. It shows that Fe^3+^ can chelate with carmine acid (Tütem et al., 1996), which changes the chromophore of carminic acid and its chelation may be stronger than Al^3+^. When the Fe^2+^ concentration was 0.001 mol/L, the solution of carminic acid was reddish‐brown and its absorption spectrum was consistent with the blank; the solution of carminic aluminum lake was purple and there were two absorption peaks at 530 nm and 570 nm in the visible light region. When the concentration of Fe^2+^ is 0.1 mol/L, the color and absorption spectrum of two colorants were consistent with that of 0.001 mol/L Fe^3+^, which may be caused by the oxidation of some Fe^2+^ in the air. After the addition of 0.001 mol/L Ca^2+^, the color and absorption spectrum of the carminic acid solution and the carminic aluminum lake solution did not change. However, when the Ca^2+^ concentration increased to 0.1 mol/L, brown flocs appeared in the carminic acid solution but the maximum absorption wavelength remained unchanged. The reason for this may be that carminic acid is an organic acid and it chelates with calcium chloride to form a water‐insoluble precipitate. Carminic aluminum lake contains both Al^3+^ and Ca^2+^ to chelate carminic acid (Harris et al., 2009; Meloan, Valentine, & Puchtler, 1971), so the further addition of Ca^2+^ has no effect on the carminic aluminum lake. For Cu^2+^, at the concentration of 0.001 mol/L, the carminic acid solution was purple and the absorption spectrum showed two absorption peaks at 540 nm and 590 nm; under the same conditions, the carminic aluminum lake solution was pink and the absorption peaks were still 530 nm and 570 nm. At high concentrations of Cu^2+^, the carminic acid solution was purple and the absorption spectrum showed two absorption peaks at 540 nm and 590 nm. The carminic aluminum lake solution was blue and the absorbance increased significantly when the wavelength was greater than 650 nm, which may be caused by the color and absorption spectrum of Cu^2+^ itself. Studies have shown that Cu^2+^ can form a bidentate ligand with carboxyl groups and ortho‐hydroxyl groups of carminic acid and its stability is stronger than the complexes formed with Zn^2+^, Ni^2+^, Co^2+^ and Hg^2+^(Atabey et al., 2012). Therefore, it is possible that the chelation of Cu^2+^ is stronger than that of Al^3+^, which lead to the color change of the carminic aluminum lake.

The above results show that Fe^3+^, Fe^2+^, and Cu^2+^ have significant effects on the color of the two colorants, limiting their application in iron‐ and copper‐containing foods. Low concentration of Ca^2+^ has no effect on carminic acid, but the color of carminic acid is significantly changed after the addition of a high concentration of Ca^2+^. It indicates that the two colorants can be used in foods with low calcium content, but its use is restricted in foods with high calcium content.

### 3.4 Effects of food additives on the colorants

**Figure 4:**
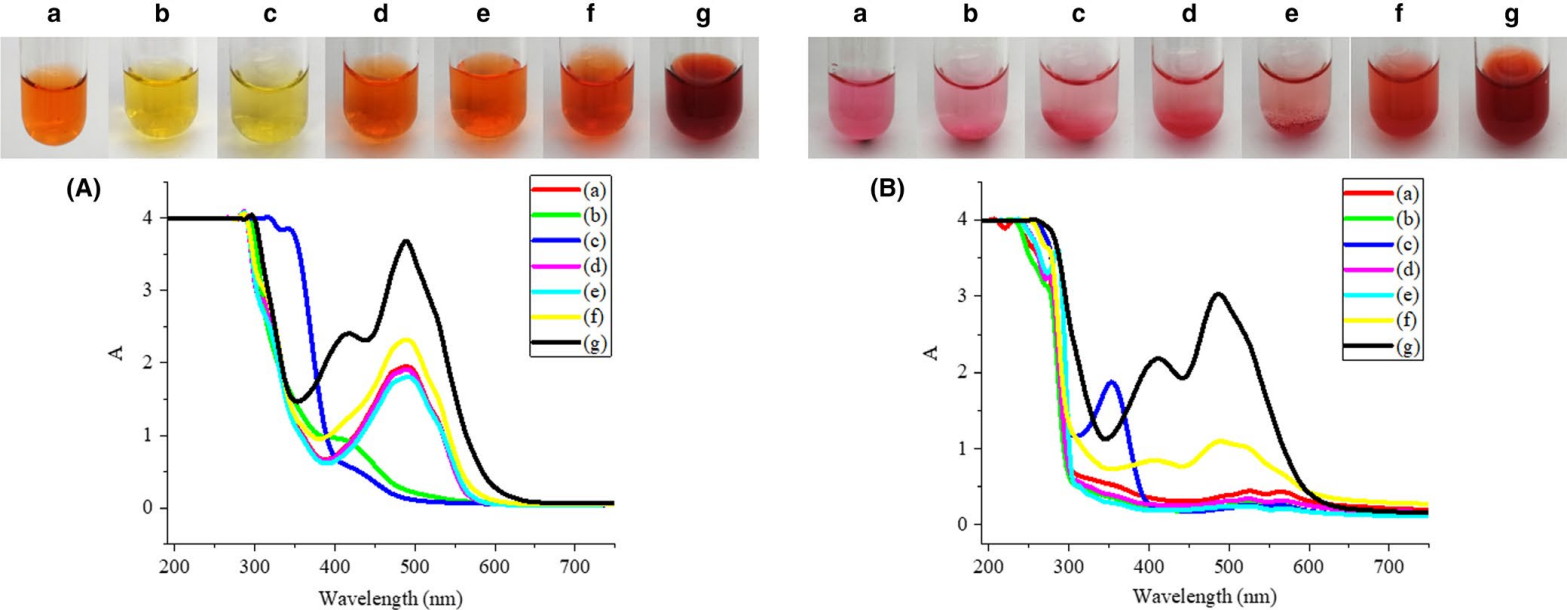
Effects of sodium nitrite, ascorbic acid, and monascus color at different concentrations on the color and absorption spectrum of carminic acid (A) and carminic aluminum lake (B). (a) Blank, (b) 0.01% sodium nitrite, (c) 1% sodium nitrite, (d) 0.01% ascorbic acid, (e) 1% ascorbic acid, (f) 0.01% monascus color, (g) 0.1% monascus color

Sodium nitrite, with its oxidizing and reducing properties, is commonly used as a chromogenic reagent and preservative in meat products. Ascorbic acid, which can reduce oxygen and protect double bonds, can be added to food as a reducing agent, antioxidant, and metal scavenger. Monascus color is commonly used as a colorant in meat products. The effects of sodium nitrite, ascorbic acid, and monascus color at different concentrations on the color and absorption spectrum of carminic acid and carminic aluminum lake are shown in Figure 4.

After adding sodium nitrite, the carminic acid solution was yellow and some bubbles were generated. Due to the acidity of the carminic acid solution, sodium nitrite may undergo an auto‐redox reaction to generate nitric oxide and nitrogen dioxide. The yellow of the carminic acid solution may be caused by dissolution of the gas. However, the color of the carminic aluminum lake was not changed by the addition of sodium nitrite. After the addition of ascorbic acid, the color and absorption spectrum of carminic acid solution and carminic aluminum lake solution kept unchanged. This suggests that the redox properties of sodium nitrite and ascorbic acid are not enough to affect the chromophore of the carminic aluminum lake. The carminic acid solution and carminic aluminum lake solution with monascus color were both red, which may be caused by the color of the monascus color. This indicates that ascorbic acid and monascus color have no effect on the color of carminic acid and carminic aluminum lake.

## 4 CONCLUSIONS

After being dyed by carminic acid, the surimi was light purple, the minced meat was red, and the milk was gray‐green; the color of carminic aluminum lake was stable, which was magenta for all the three foodstuffs. Due to the presence of amino groups in protein, MFP, WPI, SPI, and casein raise the pH of the solution, so that the color of the carminic acid changed with the pH; the color of carminic aluminum lake was magenta and not affected by protein. Fe^3+^, Fe^2+^, Cu^2+^, and Ca^2+^ at high concentrations had a great effect on the color of carminic acid and carminic aluminum lake, which limits their application in iron‐, copper‐, and high‐calcium foods. Sodium nitrite has a significant effect on the color of carminic acid, while ascorbic acid and monascus color have no significant effect on the color of carminic acid and carminic aluminum lake. These results would be helpful in providing guidance for the reasonable application of carminic acid and carminic aluminum lake in different food.

## CONFLICT OF INTEREST

The authors declare no conflict of interest.
